# *Campylobacter* Bacteriophage Cocktail Design Based on an Advanced Selection Scheme

**DOI:** 10.3390/antibiotics11020228

**Published:** 2022-02-10

**Authors:** Severin Michael Steffan, Golshan Shakeri, Corinna Kehrenberg, Elisa Peh, Manfred Rohde, Madeleine Plötz, Sophie Kittler

**Affiliations:** 1 Institute for Food Quality and Food Safety, University of Veterinary Medicine Hannover, Foundation, Bischofsholer Damm 15, 30173 Hannover, Germany; severin.michael.steffan@outlook.com (S.M.S.); elisa.peh@tiho-hannover.de (E.P.); madeleine.ploetz@tiho-hannover.de (M.P.); 2 Department of Food Hygiene and Aquaculture, Faculty of Veterinary Medicine, Ferdowsi University of Mashhad, Azadi Square, Mashhad 9177948974, Iran; golshan.shakeri@gmail.com; 3 Institute for Veterinary Food Science, Justus-Liebig-University Giessen, Frankfurter Straße 92, 35392 Giessen, Germany; corinna.kehrenberg@vetmed.uni-giessen.de; 4 Central Facility for Microscopy, Helmholtz Centre for Infection Research GmbH, Inhoffenstraße 7, 38124 Braunschweig, Germany; mcfrohde@t-online.de

**Keywords:** bacteriophages, pathogenic bacteria, phage resistance, *Campylobacter*, food safety, phage cocktail, phage selection, cocktail design, firehammervirus, fletchervirus

## Abstract

Campylobacteriosis is a worldwide-occurring disease and has been the most commonly reported zoonotic gastrointestinal infection in the European Union in recent years. The development of successful phage-based intervention strategies will require a better understanding of phage–bacteria interactions to facilitate advances in phage cocktail design. Therefore, this study aimed to investigate the effects of newly isolated group II and group III phages and their combinations on current *Campylobacter* field strains. A continuous workflow for host range and efficiency of plating (EOP) value determination was combined with a qPCR-based phage group identification and a liquid-based planktonic killing assay (PKA). An advanced analysis scheme allowed us to evaluate phage cocktails by their efficacy in inhibiting bacterial population growth and the resulting phage concentrations. The results of this study indicate that data obtained from PKAs are more accurate than host range data based on plaque formation (EOP). Planktonic killing assays with *Campylobacter* appear to be a useful tool for a straightforward cocktail design. Results show that a group II phage vB_CcM-LmqsCP218-2c2 and group III phage vB_CjM-LmqsCP1-1 mixture would be most promising for practical applications against *Campylobacter coli* and *Campylobacter jejuni*.

## 1. Introduction

*Campylobacter* enteritis (campylobacteriosis) is a widespread infectious disease in humans worldwide, most often associated with the species *Campylobacter (C.) coli* and *jejuni* [1,2]. The European Food Safety Authority (EFSA) started issuing annual summary reports on the trends and sources of zoonoses in 2005. Ever since, campylobacteriosis has been at the top of the list regarding case numbers [1]. Symptoms include watery to hemorrhagic diarrhea and abdominal pain, while post-infection, severe but rare long-term sequelae can occur, such as Guillain-Barré syndrome, reactive arthritis, and erythema nodosum [2,3,4].

*C. jejuni* and *C*. *coli* are commensals occurring in the intestines of various wild, domestic, and farm animals [1,5]. During slaughter and processing, the pathogens can contaminate animal carcasses, while later contamination of other foodstuffs by cross-contamination is possible during food preparation [6,7,8,9]. The minimum infectious dose for *Campylobacter* sp. is estimated to be below 500 colony-forming units (CFU) [10,11]. If *Campylobacter* loads in chicken ceca could be reduced by 10^2^ CFU, current models by EFSA predict a 42% relative risk reduction in campylobacteriosis attributable to the consumption of European broiler meat [1,12]. Advanced hygiene measures or feed and drinking water additives could achieve a reduction in *Campylobacter* loads [12]. However, the use of bacteriophages (phages), viruses infecting bacterial cells, could be an effective and ecological alternative to these measures [13]. A mixture of multiple phages, also known as a phage cocktail, has the advantage of broadening the overall range of bacteria susceptible to phage infection, while potentially reducing the number of bacterial cells showing reduced susceptibility to the phages [14]. Whether phage combinations could have additional advantages such as producing additive and synergistic effects or could lead to indifferent or antagonistic effects is currently not well understood. However, in recent years, an increasing number of studies have been published on this subject [14,15,16]. In addition, efforts have been made to standardize phage characterization, resulting in the improved comparability of results from different research groups [14,17,18].

Most *Campylobacter* phages specifically infect certain *Campylobacter* strains and do not cause dysbiosis in the chicken gut microbiota [19]. *Campylobacter* phages are nonenveloped viruses with a head–tail structure belonging to the order of *Caudovirales* [20]. Their icosahedral head contains AT-rich and double-stranded genomic DNA. Most *Campylobacter* phages have a contractile tail and belong to the family *Myoviridae*, while some members of the family *Siphoviridae*, with flexible tails, have been described but are not yet officially recognized by the International Committee on Taxonomy of Viruses (ICTV) [21]. The *Campylobacter* phages are further subdivided into the two genera Fletcherviruses and Firehammerviruses, based on DNA sequence analysis. These genera overlap with groups of a former typing scheme based on genome size (Firehammerviruses ≡ group II ~ 184 kb; and Fletcherviruses ≡ group III ~ 138 kb) [20,22]. Group II phages infect *C. coli* and *C*. *jejuni* and recognize their hosts via the flagellum, while group III phages are restricted to infecting *C. jejuni* and bind to their host’s capsular polysaccharides [23,24]. In the case of *Campylobacter*, a phage cocktail including group II and III phages is considered to be the best choice for targeting *C. jejuni* as well as *C*. *coli* in cases where the colonizing species is not identified [25].

Currently, only a limited number of in vivo or in vitro studies have described *Campylobacter* phages sufficiently for group assignment, making cross-study comparison difficult [26,27]. Furthermore, the first commercially available *Campylobacter* phage cocktail (CampyShield^TM^) produced by Intralytix received ‘Generally Recognized as Safe’ status as a food additive (GRAS Notice No. 966) from the United States Food and Drug Administration [28]. However, the choice of phages was documented as being based on a *C. jejuni* panel in combination with a “standard plaque assay” only [28,29,30,31]. The cocktail comprises a limited number of three phages, but its approval allows for the expansion of the cocktail to eight phages [28]. This demonstrates the need for advances in phage characterization and choice, enabling scientists and companies to modify the size and host range of phage cocktails in a real-time approach, constantly adapting it to the changing epidemiological situation. The present study is therefore an initiative to include advanced analytical methods in current *Campylobacter* phage cocktail design, with special emphasis on cocktails combining phages of different groups.

## 2. Materials and Methods

### 2.1. Bacterial Strains and Growth Conditions

The bacteria panel has been described previously by Steffan et al. [32]. In short, the panel consisted of two type strains (DSM 4688, DSM 4489), three reference strains (NCTC 11168, NCTC12662 and ATCC BAA-2151), and 24 field isolates from chicken samples collected in Lower Saxony, Germany in 2017 from commercial poultry farms, including the *C*. *coli* field strain 084610 that was used for phage isolation and as reference to calculate EOP values. Field isolates were characterized by *flaA*-typing and SmaI-PFGE macrorestriction analysis. Stock cultures of *Campylobacter* cells were stored at −80 °C. Bacterial culturing was performed on Columbia Agar sheep Blood ‘Plus’ plates (Thermo Fischer Scientific Oxoid Deutschland GmbH, Wesel, Germany) at 42 °C and under microaerobic conditions (5% O_2_, 10% CO_2_, 85% N_2_, >80% humidity). Brain–heart infusion broth (Carl Roth GmbH and Co. KG, Karlsruhe, Germany, X916) supplemented with 1 mM Calcium chloride (CBHI) served as medium for liquid cultures.

### 2.2. Bacteriophage Isolation and Propagation

Bacteriophage isolation and propagation were performed as described by Steffan et al. [32]. In brief, fecal or cecal content from Lower Saxony poultry farms was dispersed in SM-buffer (100 mM NaCl, 8 mM MgSO_4_, 50 mM Tris-HCl (pH 7.5)) using an Ultra-Turrax T10 homogenizer (IKA-Werke GmbH and Co. KG, Staufen, Germany). Skin samples were transferred into plastic bags containing SM-buffer and were thoroughly rinsed by massaging. Bacteriophages were separated from solid contaminants by two centrifugation steps and subsequent filtration of the supernatant through a 0.2 µm polyethylensulfon membrane (PES) syringe filter (Carl Roth GmbH and Co. KG, Karlsruhe, Germany). The presence of phage virions was then confirmed by coculturing the filtrate with *C. coli* field strain 084610 in Sodium-NZamines–Casamino-acids–Yeast–Magnesium-sulfate (NZCYM)–soft-agar overlay containing 0.4% agar–agar. A successive three-fold picking and plating procedure was performed to purify the phages. Phage suspensions with increasing volume and concentration were produced by switching to the bacterial host *C. coli* NCTC 12667 [20] for overlay production and washing phage particles with SM-buffer from NZCYM–soft agar overlays containing 0.7% agar–agar. Phage concentrations were determined by serial dilution of the phage lysate and duplicate plating 100 µL of each dilution in overlays containing *C. coli* NCTC 12667.

### 2.3. Phage Characterization

#### 2.3.1. Host Range/EOP

The host range of the phages was determined by a Direct Spot Test (DST) assay combined with phage dilution series, which was based on a modified method of Korf et al. [33] and previously used by Steffan et al. [32]. In short, NZCYM–soft-agar overlays were inoculated with one of 28 *Campylobacter* isolates or strains. The overlays were poured onto agar-base plates. After solidification, 10 µL of 10-fold serially diluted phage suspensions containing one of 18 phages were applied onto the overlays. After an incubation period of 20 ± 2 h, plates were inspected for plaques or opaque lysis zones. Plaque formation on *C. coli* field strain 084610 served as reference for EOP calculation and phage/bacterial combinations that produced visible plaques two to three times or visible plaques once and opaque lysis zones two to three times were used to calculate the efficiency of plating (EOP). If included, opaque lysis zones were counted as one plaque. Visible plaques or opaque inhibition zones without plaques and no lysis in the other two replicates were counted as negative results.

#### 2.3.2. Phage Classification

##### Negatively Stained Virions for Electron Micrographs

Thirty microliters of the phage solution was placed on a cover slip and a small piece of mica was floated for 15–30 s on the phage solution, washed in TE buffer (10 mM Tris, 1 mM EDTA, pH 6.9), and subsequently negatively stained with 2% aqueous uranyl acetate following the method by Valentine et al. [34]. After collecting the piece of mica with a 300-mesh copper grid, grids were blotted dry on filter paper and air-dried. Samples were examined in a Zeiss TEM 910 transmission electron microscope (Carl Zeiss AG, Oberkochen, Germany) at an acceleration voltage of 80 kV and at calibrated magnifications with a line replica. Images were recorded digitally with a Slow-Scan CCD-Camera (ProScan, 1024 × 1024, Proscan Elektronische Systeme GmbH, Scheuring, Germany) using ITEM-Software (Olympus Soft Imaging Solutions GmbH, Münster, Germany).

##### DNA-Based Analysis

Pulsed-field gel electrophoresis (PFGE)-based genome length estimation as well as macrorestriction analysis using HhaI and SwaI were performed as previously described [35]. Real-time PCR (qPCR) based on a protocol by Jäckel et al. [36] aided in assigning phages to the groups. PCR primer targets were the tail tube gene, ORF186 of CP21 (CPGII-probe: FAM-CCGGATTGACTGTAGAAACA-BHQ-1; group II phages), and the base plate wedge gene, OFR008 of CP81 (CPGIII-probe Cy5-TGTAACTGCCCTGTTTGCTG-BBQ-650).

#### 2.3.3. Phage Tests in Liquid Culture

##### Planktonic Killing Assay (PKA)

Liquid cultures of three *Campylobacter* field isolates were exposed to bacteriophages and incubated in a Tecan Spark Multiplate reader for 24 h, as described by Steffan et al. [32]. The growth of *C. jejuni* Cj18, LH83 and *C. coli* Cc4 was monitored by optical density measurements at 600 nm (OD_600_). Three group II phages (vB_CcM-LmqsL1/2, vB_CcM-LmqsL218-2c2, and vB_CcM-Lmqs288/3) and one group III phage (vB_CjM-Lmqs1-1) were used during these experiments. Phages were added at the start of the experiment using a multiplicity of infection (MOI_input_) of 10 or 0.001 (overall phage concentrations of 10^8^ or 10^4^ PFU/mL in the microplate). Either one, two, or four phages were applied simultaneously. The wells of a 48-well microplate were either filled with (i) 500 µL CBHI as optical background control, (ii) 250 µL bacteria suspension + 250 µL CBHI for bacterial growth standard (control curve), or (iii) 250 µL bacteria suspension + 250 µL phage suspension for analysis of bacterial reduction (treatment curve). Plates were incubated with double orbital shaking under microaerobic conditions (5% O_2_, 10% CO_2_, 85% N_2_, 108 rpm) at 42 °C. Experiments were performed in quadruplicate with single well replication per plate to receive a minimum of three evaluable replicates for all experiments.

The optical background was subtracted from initial OD_600_ measurements, and the area under the curve (AUC) values were determined by spline fitting these adjusted results (control and treatment curves) from start- to endpoint of the experiments (0 to 24 h). AUC values of single-phage PKAs were used for statistical analysis (Dunnett’s test). Mean AUC values of all PKAs (A_i_) were used to calculate virulence indices (v_i_) [17] for both MOI_input_ levels (10 and 0.001) and to combine the results into a mean virulence index for both MOI_input_ levels (mean_vi_) (see Equation (1)).

(1) $$mean_{vi}=\frac{\overset{v_{i}forMOI_{input}10}{\overbrace{\left(1-\frac{A_{i10}}{A_{control}}\right)}}+\overset{v_{i}forMOI_{input}0.001}{\overbrace{\left(1-\frac{A_{i0.001}}{A_{control}}\right)}}}{2}$$

##### Comparison of Phage Concentrations after 24 h

At the end of the PKA (24 h), half of the remaining content in the wells was withdrawn, and the phages were separated by pelleting the bacteria by centrifugation (16,000× *g*, 3 min) and filtrating the supernatant using a 0.2 µm PES syringe filter. Subsequently, phage concentrations were determined as described above using *C. coli* NCTC 12667 as host bacterium for the group II phages, and *C. jejuni* NCTC 12662 as host for the group III phage. The concentration of group II phages that were applied in multi-group-II-phage PKAs could not be determined separately, as they shared the same host. Thus, sum parameters were calculated for these PKAs. To confirm that group II and group III phage concentrations from multi-phage PKAs with both groups could be determined individually, the absence of plaque formation on the other detection strain (group II phages on NCTC 12662 and the group III phage on NCTC 12667) was confirmed four times by DST assay using a phage concentration of 10^8^ PFU/mL. The absence of plaques was confirmed, except for CP218-2c2 and *C*. *jejuni* NCTC 12662 (10^2^ to 10^3^ PFU/mL).

The mean phage concentration after 24 h (c_j_) was divided by the mean phage concentrations at the start of the experiment (c_0_) to calculate the adjusted concentration (c_adj_). For multi-phage PKAs, a mean of all single-phage concentrations was used as the starting concentration (c_0_) (see Table 1). Adjusted concentrations for both MOI_input_ levels were used to calculate the mean adjusted phage concentration (mean_c24_) according to Equation (2).

(2) $$mean_{c24}=\frac{\overset{c_{adj}forMOI_{input}10}{\overbrace{\left(\frac{c_{j10}}{c_{0}}\right)}}+\overset{c_{adj}forMOI_{input}0.001}{\overbrace{\left(\frac{c_{j0.001}}{c_{0}}\right)}}}{2}$$

### 2.4. Data Analysis

Data preparation, visualization, and statistical analysis (Dunnett’s test) were performed with R software (version 4.1.0) including the packages DescTools (version 0.99.42), dplyr (version 1.0.7), ggeasy (version 0.1.3), ggplot2 (version 3.3.5), ggrepel (0.9.1), gridExtra (version 2.3), and gtable (version 0.3.0). The host range/EOP heat map was generated using the ComplexHeatmap package (Gu, Eilis, and Schlesner, 2016), while ImageJ version 1.51q in the Fiji bundle in combination with the ObjectJ plugin was used to determine phage particle size parameters.

## 3. Results

### 3.1. Phage Isolation and Host Range Determination

*Campylobacter-coli*-specific phages were isolated from a previously described sample set (*n* = 301; *n*_cecal_ = 136, *n*_fecal_ = 111, *n*_neck skin_ = 54) [32]. The *C. coli* field strain 084610 was used as indicator bacterium for detecting phages. In total, 18 phages were isolated from chicken feces (*n* = 4), cecal content (*n* = 2), and neck skin samples (*n* = 4) in 2017. Some samples yielded more than one phage, as shown in Table 1. In addition, three phages deriving from chicken samples were provided by a clinical laboratory. No additional information was given on isolation source or time of these phages.

**Figure 1:**
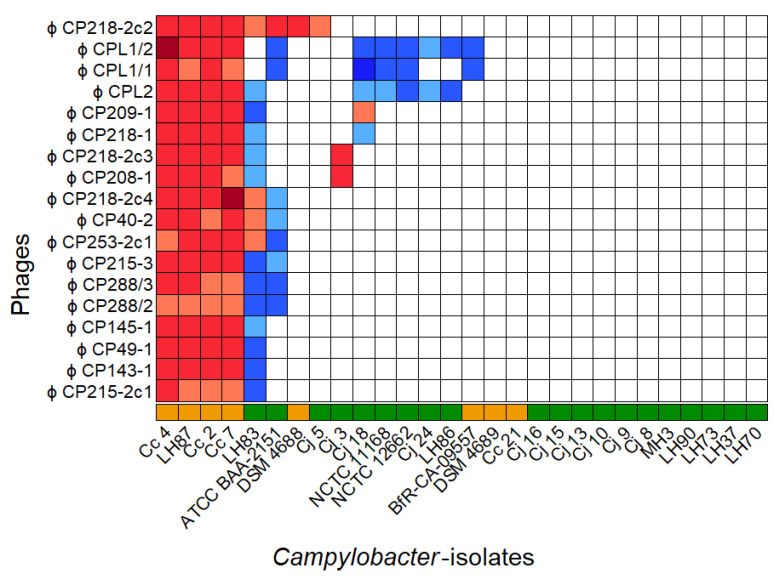
Host range of 18 *C. coli*-specific phages on 28 *Campylobacter* isolates with color-coded EOP values. Host range evaluation was performed by spotting serial dilutions of bacteriophages onto NZCYM–soft-agar overlays containing the respective bacterial isolate (◼ *C*. *jejuni*, ◼ *C*. *coli*). Experiments were performed in triplicate. *C. coli* 084610 was used as reference strain, and color coding was used to visualize EOP values  (◼  x>1.2,  ◼  1.2≥x>1,  ◼  1≥x>0.9,  ◼  0.9≥x>0.8,  ◼  0.8≥x>0.4,  ◼  0.4≥x>0,  □x=0).

The host range and EOP values for all 18 phages were determined by a DST assay combined with phage dilution series. The panel consisted of 28 *Campylobacter* isolates, and *C. coli* field strain 084610 served as reference for EOP calculation [32]. No reproducible plaque formation was observed on two *C. coli* and eleven *C. jejuni* isolates, while six *C. coli* and nine *C. jejuni* were susceptible to at least one phage as shown in Figure 1. 

Of the 18 phages, vB_CcM-LmqsCPL1/2 (CPL1/2), vB_CcM-LmqsCP218-2c2 (CP218-2c2), and vB_CcM-LmqsCP288/3 (CP288/3) were selected for further characterization. In addition, the undescribed phage vB_CjM-Lmqs1-1 (CP1-1), from a previous isolation scheme, was added [32]. These phages were chosen based on their broad and differing host ranges, including a panel of two C. *jejuni* (LH83, Cj18) field strains and one *C. coli* (Cc4) field strain that were used for further experiments (see Table 2). The selected subset of *Campylobacter* isolates and phages was the prerequisite for the following PKA tests.

### 3.2. Phage Classification

**Figure 2:**
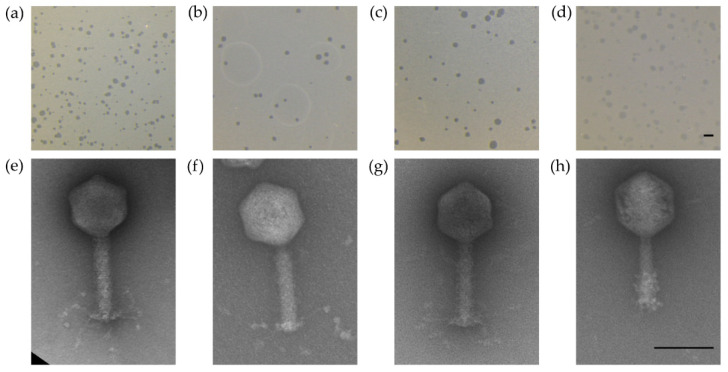
Plaque morphology of the four examined myovirus phages (**a**–**d**) and micrographs showing the morphology of the virions (**e**–**h**). All four phages, CPL1/2 (**a**,**e**), CP218-2c2 (**b**,**f**), CP288/3 (**c**,**g**), and CP1-1 (**d**,**h**), formed clear plaques on *C. coli* strain NCTC 12667 (**a**–**c**) and *C. jejuni* strain NCTC 12662, respectively (**d**); scale bar represents 2 mm. The virions consisted of an icosahedral head and a contractile tail structure with tail fibers; negatively stained phage particles with 2% uranylacetate; scale bar represents 100 nm.

The four chosen phages formed clear plaques on their respective host strains (CPL1/2, CP218-2c2, and CP288/3 on *C. coli* NCTC 12667; CP1-1 on *C. jejuni* NCTC 12662) as shown in Figure 2a–d. Plaque diameters ranged from 0.64 to 1.13 mm (Table 3). Negatively stained electron micrographs showed virions with a head–tail structure as is common for members of the *Myoviridae* family (Figure 2 e–h). All of the four phages showed a similar head size, while the tail structures of CPL1/2 and CP1-1 were shorter than those of CP218-2c2 and CP288/3 (Table 3). Genome comparison by PFGE revealed a smaller genome for CP1-1 (~145 kb), while the group II phage genomes of CPL1/2, CP218-2c2, and CP288/3 appeared to be of equal size (~175 kb) (Table 3). Further genomic differences were identified after exposure to the restriction endonucleases HhaI (5’…GCGꜜC…3’) and SwaI (5’…ATTTꜜAAAT…3’). The DNA of CP1-1 was susceptible to digestion by HhaI, while the group II phage DNA was resistant to HhaI and susceptible to SwaI (Table 3). Based on the abovementioned and additional qPCR results, CPL1/2, CP218-2c2, and CP288/3 were classified as group II phages, and CP1-1 was classified as a group III phage. 

### 3.3. Phage Testing in Liquid Culture

#### 3.3.1. Planktonic Killing Assay (PKA)

Liquid cultures of *C. jejuni* Cj18 and LH83 as well as *C. coli* Cc4 were incubated in the presence and absence of phages and growth was monitored by optical density measurements at OD_600_ using a Tecan Spark Multiplate reader for 24 h. Phages were added at the start of the experiment at MOI_input_ 10 (high) or MOI_input_ 0.001 (low), and as either one-, two-, or four-phage applications. The examined combinations of bacteria and phages were chosen based on the previous host range/EOP analysis. The chosen combinations included a broad range of EOP values (see Table 2) and phages with different host ranges [14].

**Figure 3:**
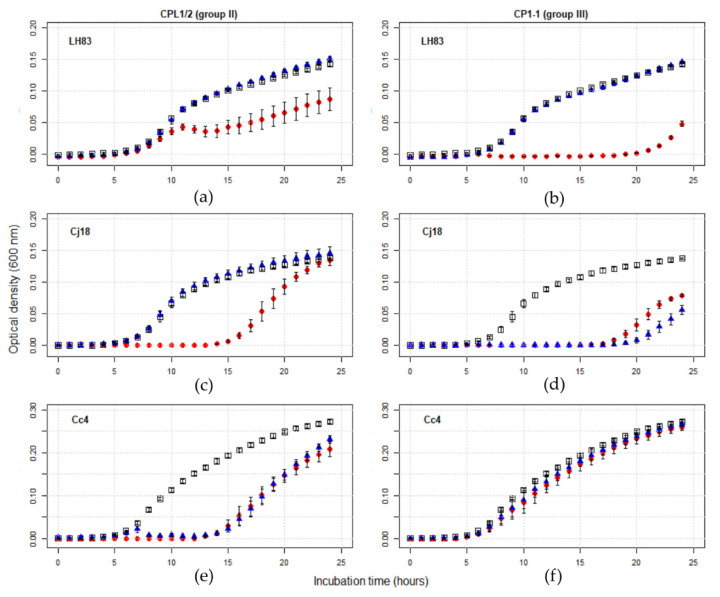
Exemplary growth curve comparison of *Campylobacter* alone and in presence of two phage concentrations. PKA experiments were performed in a Tecan Spark Microplate Reader. Optical density was measured every hour at 600 nm for 24 h. Curves represent mean OD_600_ values of three or four experiments, with error bars indicating standard error of the mean. Control curves (□) and PKA curves for MOI_input_ 10 (●) and 0.001 (▲) are displayed. The observed effects of phages on bacterial population growth could be classified into three groups: either no effect at all (e.g., Cc4 + CP1-1 (**f**)), limited growth impediment at a high MOI_input_ only (e.g., LH83 + CPL1/2 (**a**) or LH83 + CP1-1 (**b**), Cj18 + CPL1/2 (**c**)), or growth was impeded at both a high and low MOI_input_ (e.g., Cj18 + CP1-1 (**d**) or Cc4 + CPL ½ (**e**)).

Of the twelve combinations containing single phages, eight produced plaques in a reproducible manner during host range/EOP tests, and effectivity for bacterial population growth impediment was confirmed by the PKA results. Of the four combinations without plaque formation, three effectively impeded bacterial population growth in liquid culture (Cj18 + CP218-2c2 and CP288/3, and LH83 + CPL1/2), while one did not (Cc4 + CP1-1). The observed phage effects on bacterial population growth could be classified into three groups (Figure 3). Either no effect at all was observed (e.g., Cc4 + CP1-1; Figure 3f), limited growth impediment occurred at a high MOI_input_ only (e.g., LH83 + CPL1/2 or LH83 + CP1-1, Cj18 + CPL1/2; Figure 3a–c), or growth was impeded, both at a high and low MOI_input_ (e.g., Cj18 + CP1-1 or Cc4+CPL1/2; Figure 3d,e). Thus, PKA results allowed us to identify effective phage/bacteria combinations that were not identified as effective by host range/EOP tests.

#### 3.3.2. Comparison of AUC Values after Single-Phage Application

**Figure 4:**
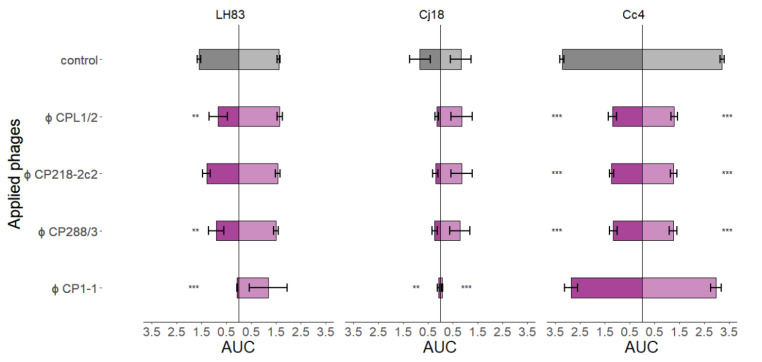
Area under the curve (AUC) values from planktonic killing assays for single-phage applications on three different *Campylobacter* strains. *C. jejuni* LH83, Cj18, and *C. coli* Cc4 were exposed to three group II phages (CPL1/2, CP218-2c2, and CP288/3) or one group III phage (CP1-1) in single-phage planktonic killing assay at an MOI_input_ 10 (■/■) or MOI_input_ 0.001 (■/■). Experiments were performed in triplicate or quadruplicate. Dunnett’s test with a 95% confidence level was used to compare the AUC values of the treatment to the control (significance code indicates range of *p* values: ** 0.001, *** > 0.001). Error bars indicate the standard error of the mean.

The area under each growth curve (AUC) was determined by spline fitting from 0 to 24 h (Figure 4). Results from single-phage PKAs were used to determine the efficacy of single phages in detail, and to form a base for interpreting the results of phage combinations. *C. jejuni* LH83 showed significantly lower AUC values than the control at high MOI_input_ PKAs, but only if the two group II phages CPL1/2 and CP288/3 or the group III phage CP1-1 were used. CP1-1 was the most effective of the three phages. In low MOI_input_ PKAs, none of the four phages resulted in reduced population growth of LH83 compared to the control. Similar results were obtained for *C. jejuni* Cj18. The three group II phages were only able to reduce bacterial population growth in high MOI_input_ PKAs. However, these results were not significant, and no difference to the control was observed at a low MOI_input_. In contrast, CP1-1 PKAs resulted in significant population growth reduction in *C*. *jejuni* LH83, independent of the MOI_input_ levels used, the same being true for group II phage PKAs using *C. coli* Cc4. Importantly, the bacterial population growth reduction in Cc4 in experiments using a low MOI_input_ was in contrast to the results from experiments using the two *C. jejuni* strains. The *C*. *coli* Cc4 had proven resistant to the group III phage CP1-1 during host range tests, as expected by the host range of group III phages, including *C*. *jejuni* only. In accordance, no reduced population growth was detected in Cc4 + CP1-1 PKAs. Reduced bacterial population growth compared to the control was observed in all low and high MOI_input_ PKAs using all three group II phages and field isolate Cc4 or the group III phage CP1-1 and field isolate Cj18.

#### 3.3.3. Comparison of Phage Concentrations after 24 h in Single-Phage PKAs

**Figure 5:**
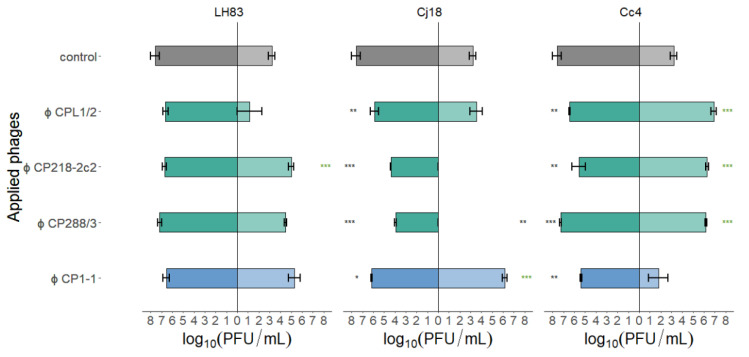
Final phage concentrations after single-phage planktonic killing assays with three different *Campylobater* field isolates. *C. jejuni* LH83, Cj18, and *C. coli* Cc4 were exposed to either one of the three group II phages (CPL1/2, CP218-2c2, and CP288/3) or the group III phage (CP1-1) at MOI_input_ 10 (■/■/■) or MOI_input_ 0.001 (■/■/■). Experiments were performed in triplicate or quadruplicate. Dunnett’s test with a 95% confidence level was used to compare the phage concentrations at the beginning of the experiments with those after 24 h (significance code indicates range of *p* values: * 0.01, ** 0.001, *** >0.001; * final concentration higher than starting concentration). Error bars indicate the standard error of the mean. Control bars represent mean starting concentrations (■/■).

At the end of each PKA, phage concentrations were determined. Phage concentrations in single-phage PKAs determined at the beginning (0 h, see Table 4) and at the end of the experiment (24 h) were used for statistical analysis (Dunnett’s test). Results are displayed in Figure 5.

In the case of the PKAs using *C. jejuni* LH83 and a high MOI_input_, final phage concentrations were equivalent to the starting concentrations. At a low MOI_input_, the final phage concentrations of CP218-2c2 were significantly increased, while the concentrations for the phages CP288/3 and CP1-1 were equivalent to the starting concentrations. However, when using CPL1/2 at a low MOI_input_, only in one of three samples single phage plaques were detected at the end of the experiment. In contrast, single-phage PKAs with Cj18 using a high MOI_input_ resulted in phage concentrations after 24 h that were significantly lower than the starting concentrations. In low MOI_input_ PKAs, however, CPL1/2 concentrations were equivalent to the starting concentrations, while CP1-1 phage concentrations were significantly elevated, and the final phage concentrations of CP218-2c2 and CP288/3 in PKAs were reduced below the detection limit. For PKAs using Cc4 and a high MOI_input_, all final phage concentrations were significantly reduced compared to the starting concentrations, while at a low MOI_input_, all group II phages significantly increased and concentrations of CP1-1 remained stable during the PKAs. Overall, phage concentrations reached values similar or below the starting concentrations when a high MOI_input_ was used, while in the case of a low MOI_input_, final phage concentrations exceeded the starting concentrations in some experiments.

#### 3.3.4. Multi-Phage PKAs

After detailed analysis of single-phage PKAs, multi-phage PKAs were conducted. These experiments were subdivided into PKAs using (i) two group II phages (CPL1/2 + CP218-2c2, CPL1/2 + CP288/3, and CP218-2c2 + CP288/3), (ii) the group III phage and one group II phage (CP1-1 + CPL1/2, CP1-1 + CP218-2c2, and CP1-1 + CP288/3), or (iii) all four phages (CPL1/2 + CP218-2c2 + CP288/3 + CP1-1). Curves resulting from PKAs using one or two group II phages appeared to have similar shapes independent of the MOI_input_ used (see Figure 3). Combining group II phages with the group III phage resulted in PKA curves having the same shape as the single-group-III-phage PKAs, when *C. jejuni* field isolates LH83 or Cj18 were used. If Cc4 was used in PKAs with these mixtures, the shape of the curves remained similar to PKAs using group II phages only. Interestingly, the growth curves from single-group-II-phage PKAs using LH83 showed an initial small peak, subsequently declining during the experiment as shown in Figure 3a. This observation was true for all group II phages/LH83 combinations except for LH83 + CP218-2c2, where both MOI_input_ treatment curves had the same shape as the control curve.

**Figure 6:**
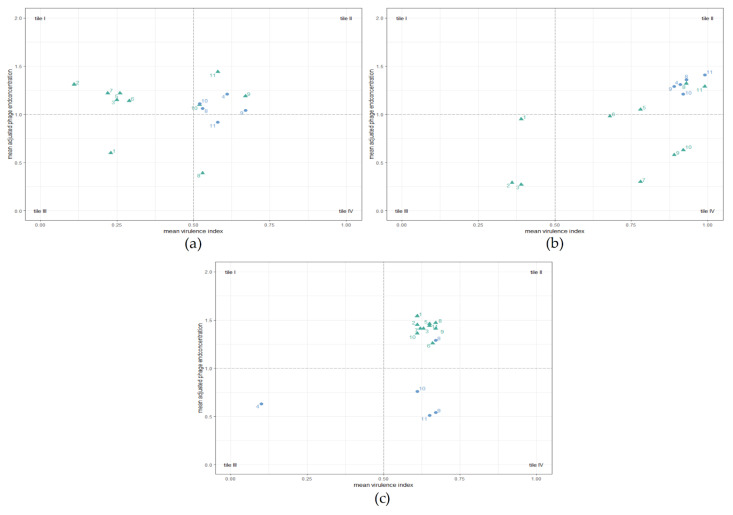
Planktonic Killing Assays (PKA): mean virulence index (mean_vi_) and mean adjusted phage concentrations after 24 h (mean_c24_). *C. jejuni* LH83 (**a**), Cj18 (**b**), and *C. coli* Cc4 (**c**) were exposed to three group II phages (CPL1/2, CP218-2c2, and CP288/3) or one group III phage (CP1-1) in one-, two-, or four-phage PKAs at MOI_input_ 10 or MOI_input_ 0.001. Experiments were performed in triplicate or quadruplicate. For all applications, the mean_vi_ and the mean_c24_ were calculated. Color coding indicates the mean_c24_ of group II phage(s) (▲) or group III phage (●). Plots were divided into four tiles. Tiles I and III represent suboptimal, while tiles II and IV represent optimal mean_vi_ values. Tiles III and IV represent suboptimal phage or no phage replication, while tiles I and II represent optimal phage replication. Number codes representing phage applications: 1—CPL1/2; 2—CP218-2c2; 3—CP288/3; 4—CP1-1; 5—CPL1/2 + CP218-2c2; 6—CPL1/2 + CP288/3; 7—CP218-2c2 + CP288/3; 8—CP1-1 + CPL1/2; 9—CP1-1 + CP218-2c2; 10—CP1-1 + CP288/3; 11—all four phages.

For further analysis and increased comparability, a mean virulence index (mean_vi_) and a mean adjusted phage final concentration (mean_c24_) were calculated for every phage combination_._ Both values were plotted against each other in a scatter plot as shown in Figure 6. The mean_vi_ can take any value between 0 and 1, while values for mean_c24_ range from 0 to 2. Thresholds were defined at a mean_vi_ of 0.5 and a mean_c24_ of 1.0. These thresholds are represented as dashed lines and divide the scatter plots into four tiles. The mean_vi_ values in tiles I and III represented suboptimal efficacy of the phages, while mean_vi_ values in tiles II and IV represented optimal efficacy. Additionally, mean_c24_ value ranges in tiles III and IV were interpreted as suboptimal and in tiles I and II as optimal phage replication ability. The concentrations for multiple group II phages were summed up, as the host *C. coli* NCTC 12667 did not allow for differentiation between group II phages.

One- and two-phage PKAs using *C. jejuni* LH83 and group II phages resulted in a mean_vi_ below the threshold (see Figure 6a, tiles I and III, nos. 1, 2, 3 and 5, 6, 7). Only mean_vi_ values from PKAs using the group III phage CP1-1 (no. 4) alone or in combination with other phages surpassed the threshold (see Figure 6a, tiles II and IV, nos. 4, 8, 9, 10, and 11). In PKAs using LH83, the mixture of CP1-1 + CP218-2c2 (no. 9) produced the highest mean_vi_ and the highest mean_c24_ values for the group II phages as well as the group III phage. However, all other PKAs using phage mixtures including CP1-1 resulted in lower mean_vi_ values than the single CP1-1 PKA (see Figure 6a, tile II, nos. 8, 11, and 10). Concerning the final phage concentrations, all mean_c24_ values were above the threshold of one, except for PKAs with LH83 using only CPL1/2, CP1-1 + CPL1/2 (only group II), or all four phages (only group III) (see Figure 6a, tile III, no. 1; and tile IV, nos. 8 and 11).

For *Campylobacter* isolate Cj18, all single-group-II-phage PKAs resulted in mean_vi_ and mean_c24_ values below the respective threshold (see Figure 6b, tile III, nos. 1, 2, and 3). Mixtures of more than one group II phage resulted in increased mean_vi_ values (see Figure 6b, tiles II and IV, nos. 5, 6, and 7), and using mixtures containing group III phage CP1-1 increased the mean_vi_ values even further, these now being similar to the single-group-III-phage PKA (see Figure 6b, tile II, nos. 8, 9, 10, and 11; single group III phage no. 4). All-phage PKAs using Cj18 (no. 11) resulted in the highest mean_vi_ and mean_c24_ values.

*C. coli* Cc4 is known to be intrinsically resistant against CP1-1 (group III), as confirmed by host range/EOP analysis and PKA results. Mean_vi_ and mean_c24_ values were low (see Figure 6c, tile III, no. 4). All PKAs containing group II phages resulted in mean_vi_ values above 0.5 and mean_c24_ values above 1, forming a cluster in tile II (see Figure 6c, nos. 1, 2, 3, 5, 6, 7, 8, 9, 10, and 11). Of these Cc4 experiments, the PKAs using CP1-1 + CPL1/2 and CP1-1 + CP218-2c2 resulted in the highest and almost identical mean_vi_ values (see Figure 6c, nos. 8, and 9). For the final phage concentrations of the multi-phage PKAs using group III phages along with group II phages, mean_c24_ values ranged below the threshold in the case of the group III phage (see Figure 6c, nos. 9, 10, and 11), but not if the mixture of CP1-1 and CPL1/2 was used (no. 8).

Overall, multi-phage PKAs with *C. jejuni* containing CP1-1 resulted in growth curves that appeared predominantly influenced by the group III phage (CP1-1), and high mean_vi_ values were calculated for these PKAs.

## 4. Discussion

The global public health burden of human campylobacteriosis raises the need for fast and efficient *Campylobacter* phage cocktail design that facilitates *Campylobacter* load reduction in the poultry meat production line. Rational cocktail design is based on host range evaluation, phage group identification, and tests for efficiency in single- and multi-phage applications (bacterial reduction, phage virion replication, etc.). This study combined a protocol for host range and EOP value determination [32] with a qPCR-based phage group identification [36] and a PKA assay that allowed the evaluation of phage combinations for *Campylobacter*-specific phage cocktail design, with an analysis scheme partially based on the virulence index proposed by Zachary et al. [17]. Phage concentrations after 24 h were included in the evaluation for phage selection [37] to ensure phage persistence during treatment [38,39]. The selected parameters aimed at identifying effective phage combinations that could potentially amplify treatment results by virion replication [37]. This workflow allowed the fast identification and characterization of new phages and enabled the identification of phage combinations that are efficient in bacterial reduction and can persist for at least 24 h [38].

Phage replication in PKAs was assumed based on the following conditions and assumptions: previous tests had shown that only a minimal decrease in phage concentration occurred in liquid media without bacteria [32]. It was assumed that a significant reduction in phage concentration was only possible if phage virions adsorbed to host cells and were either not at all or insufficiently replaced by phage replication. A phage concentration after 24 h equivalent to the starting concentration could indicate no phage virion adsorption or a steady state between adsorption and replacement by replication, while a significant increase in phage concentration after 24 h would mean that phage virion replication exceeded the loss of phage particles. Plaque formation of all phages on opposite host strains (group II phages on NCTC 12662 and group III phage on NCTC 12667) was tested four times by DST assay with the highest concentration that it was possible to produce (10^8^ PFU/mL). No plaque formation could be observed except for CP218-2c2 on NCTC 12662. As plaque formation on NCTC 12662 was reduced by 5 to 6 log units compared to NCTC 12667, this was considered a negligible problem.

Similar to Haines et al. [14], a set of phages (groups II and III) as well as a set of bacteria (*C. coli* and *C. jejuni*) were chosen for the PKA experiments in our study, which covered different EOP values. The host range of the twelve combinations used comprised eight that produced plaques during EOP/host range tests. For these combinations, bacterial reduction was confirmed during PKA. However, of the four combinations forming no plaques, three reduced bacterial growth in PKAs. Previous studies also reported differences between PKA and host range results. In contrast to our results, Haines et al. [14,18] reported that more effective phages were identified against *Klebsiella* based on EOP analysis compared to PKA, while results varied in studies for *E*. *coli*. Comparison of the EOP values with the AUC values of single-phage PKAs in our study did not reveal a relationship in the efficacy indicated by both methods (see Figure 4) or of phage concentrations after 24 h in single-phage PKAs compared to AUC values. However, EOP values showed a possible relationship with phage concentrations after 24 h in low-MOI_input_ PKAs (see Figure 5). If EOP values were zero, phage concentrations after 24 h were also low or below the detection limit, while EOP values of 0.9 were associated with final PKA phage concentrations significantly higher than the starting concentrations. In high-MOI_input_ PKAs combining the phage CP1-1 and *C*. *jejuni* Cc4, we observed a significant reduction in phage concentration after 24 h. This reduction was slightly greater than expected for losses resulting from phage degradation by temperature and/or potential pH shifts [32]. The reasons for this observation remain unknown. The potential relationship between EOP values and phage concentrations is not surprising, as EOP is a relative measure of the phages’ ability to form plaques. However, phage cocktail efficacy could be limited if phage mixtures are designed based on EOP results only [28,39].

As the efficacy of individual bacteriophages does not necessarily translate into efficient phage cocktails [40], the analyses of one-phage PKAs were followed up by multi-phage PKAs (see Figure 6). The introduction of the parameters mean_vi_ and mean_c24_ allowed for a streamlined analysis of the results. In the case of *C. jejuni* field strains (LH83 and Cj18), all single-group-II-phage PKAs produced suboptimal results (see Figure 6). A combination of multiple group II phages was able to increase the mean_vi_ values to optimal levels on field strain Cj18; this, however, was not the case when using LH83 (see Figure 6a–b). *C. coli* Cc4 group-II-phage PKAs always resulted in optimal mean_vi_ values, with low variance between different combinations (see Figure 6c). In combination with the two *C. jejuni* field strains, mixtures of group II phages and the group III phage increased the mean_vi_ values above the values in the group-II-phage PKAs. In the case of PKAs using Cc4, CP1-1 + CPL1/2 and CP1-1 + CP218-2c2 produced the highest mean_vi_ values. As all of these combinations contained the group III phage, it appeared as if the combination of group II phages and the group III phage had a positive effect on the mean_vi_. However, the mean_vi_ values for all PKAs using field isolate Cc4 were very similar, and thus the differences should not be overinterpreted. Additional studies are required to understand the mechanisms of phage interaction in these combinations. The phage combinations with the highest mean_vi_ values were CP1-1 + CP218-2c2 in the case of LH83, all four phages in the case of Cj18, and CP1-1 + CPL1/2 as well as CP1-1 + CP218-2c2 for Cc4.

Synergistic effects (see Figure 6b, no. 11 of all phages) or antagonistic effects (see Figure 6a, no. 8, CP1-1 + CP1/2; and no. 11 of all phages) on the mean_c24_ were observed in PKAs combining group II phages and the group III phage. Of particular interest were the results of PKAs applying CP1-1 + CPL1/2 on Cc4 (see Figure 6c, tile II, no. 8). The mean_c24_ values for phages of both groups surpassed the threshold of one, while the mean_c24_ values of the group III phage in one- and two-phage PKAs remained below the threshold. The reasons for this observation are unclear. Combining PKA mean_vi_ results with mean_c24_ phage concentrations led to the selection of a cocktail containing group II phage CP218-2c2 and group III phage CP1-1 for future application tests. A mixture of both phages produced high mean virulence index in PKAs with all three *Campylobacter* field strains. In addition, high mean_c24_ values indicated that CP218-2c2 virions could replicate in LH83 and Cc4 cells, while the same appeared to be true for CP1-1 in Cj18.

## 5. Conclusions

Results from this and previous studies [14,17,18,32] indicate that the formulation of phage cocktails in general could profit from an increased use of advanced selection schemes incorporating methods such as planktonic killing assays (PKAs), which could be combined with standardized analysis methods. This study describes a continuous workflow for host range and EOP value determination [32] in combination with a qPCR-based phage group identification [36] and a PKA assay that allowed us to evaluate phage cocktails with an advanced analysis scheme partially based on the virulence index proposed by Zachary et al. []. Host range/EOP values were the general basis for the initial phage selection. However, the results from this study indicate that data obtained from liquid-based assays (e.g., PKA) are more accurate for identifying host infection by phages. Combining group II and group III phages and incorporating phage concentration analysis into the scheme could help to identify phage cocktails preventing bacterial phage-resistance. It appears reasonable in the light of *Campylobacter* phage cocktail development to continue with PKA assays and to anticipate phage resistance as described by Gu et al. [41].

## Figures and Tables

**Table 1:** Isolated phages, sample origin, and year of isolation.

No.	Phage	Isolation Source	Year
1	CP40-2	caeca	2017
2	CP49-1	skin	2017
3	CP143-1	skin	2017
4	CP145-1	skin	2017
5	CP208-1	feces	2017
6	CP209-1	feces	2017
7	CP215-2c1 ^a^	caeca	2017
8	CP215-3 ^a^	caeca	2017
9	CP218-1 ^b^	skin	2017
10	CP218-2c2 ^b^	skin	2017
11	CP218-2c3 ^b^	skin	2017
12	CP218-2c4 ^b^	skin	2017
13	CP253-2c1	feces	2017
14	CP288/2 ^c^	feces	2017
15	CP288/3 ^c^	feces	2017
16	CPL1/1	n/a	n/a
17	CPL1/2	n/a	n/a
18	CPL2	n/a	n/a

Phages originated from the same sample: ^a,b,c.^ Information not available: n/a.

**Table 2:** EOP values for the phage–bacteria combinations used during PKA tests.

*Campylobacter* Field Isolate	EOP Values of *Campylobacter* Phages			
	CPL1/2	CP218-2c2	CP288/3	CP1-1
*C. jejuni* LH83	0	0.9	0.6	0.6
*C. jejuni* Cj18	0.4	0	0	1.0
*C. coli* Cc4	1.3	1.0	1.0	0

**Table 3:** Morphologic and genomic characteristics of the examined bacteriophages. Genome size and HhaI and SwaI restriction sensitivity were determined based on PFGE analysis, virion dimensions based on electron micrographs, and mean plaque diameters based on macrographs (± variance).

*Campylobacter* Phage				
	CPL1/2	CP218-2c2	CP288/3	CP1-1
Genome length by PFGE (kb)	~175	~175	~174	~145
HhaI sensitivity	−	−	−	+
SwaI sensitivity	+	+	+	not tested
**Dimensions Based on Electron Micrographs (*n* = 5)**				
Mean tail length (nm)	122.49 ± 5.01	133.46 ± 2.19	139.18 ± 1.56	112.21 ± 1.21
Mean head diameter (nm)	97.49 ± 2.61	91.41 ± 3.58	101.04 ± 8.41	97.79 ± 2.4
Mean head length (nm)	103.2 ± 8.05	108.28 ± 8.01	104.86 ± 3.88	108.34 ± 2.76
Virus family	*Myoviridae*	*Myoviridae*	*Myoviridae*	*Myoviridae*
qPCR	group II	group II	group II	group III
**Mean Plaque Diameter (24 h, 0.7% overlay, *n* = 200) (mm)**				
	0.64 ± 0.07	0.75 ± 0.08	0.93 ± 0.1	1.13 ± 0.34

**Table 4:** Mean phage concentrations (c_0_) at the beginning of single-phage planktonic killing assays (log_10_(PFU/mL)).

*Campylobacter* Phage	Starting Concentrations c_0_ (log_10_(PFU/mL))	
	MOI_input_ 10	MOI_input_ 0.001
CP1-1	7.2 ± 0.1 *	3.5 ± 0.0 **
CPL/1/2	8.2 ± 0.8 *	3.0 ± 0.2 **
CP218-2c2	7.6 ± 0.6 *	2.9 ± 0.2 **
CP288/3	7.3 ± 0.1 *	3.4 ± 0.1 **

The means of values marked with * or ** were calculated and added to Figure 5 as visual aids (control).

## Data Availability

All data available is included in the submission of the manuscript.
